# A brain slice experimental model to study the generation and the propagation of focally-induced epileptiform activity

**DOI:** 10.1016/j.jneumeth.2015.04.001

**Published:** 2016-02-15

**Authors:** Gabriele Losi, Iacopo Marcon, Letizia Mariotti, Michele Sessolo, Angela Chiavegato, Giorgio Carmignoto

**Affiliations:** Neuroscience Institute, National Research Council (CNR) and Department of Biomedical Sciences, University of Padova, via U. Bassi 58/b, 35121 Padova, Italy

**Keywords:** Focal seizures, Temporal cortex, GABAergic interneurons, Astrocytes

## Abstract

The early cellular events that in a brain network lead to seizure generation and govern seizure propagation are probably based on different cellular mechanisms. Experimental models in which these events can be separately studied would contribute to improve our understanding of epilepsy. We recently described an in vitro model in entorhinal cortex slices from young rats in which focal seizure-like discharges (SLDs) can be induced in spatially defined regions and at predictable times by local NMDA applications performed in the presence of 4-amimopyridine (4-AP) and low extracellular Mg^2+^. Through the use of single-dual cell patch-clamp and field potential recordings, and Ca^2+^ imaging from large ensembles of neurons, interneurons and astrocytes, we here extend this model to entorhinal and temporal cortex slices of rat and mouse brain, providing evidence that multiple SLDs exhibiting the typical tonic–clonic discharge pattern can be also evoked in these cortical regions by successive NMDA applications. Importantly, the temporal cortex is more accessible to viral vector injections than the entorhinal cortex: this makes it feasible in the former region the selective expression in inhibitory interneurons or principal neurons of genetically encoded Ca^2+^ indicators (GECI) or light-gated opsins. In this model, an optogenetic approach allows to activate specific neuronal types at spatially defined locations, i.e., the focus or the propagating region, and at precise time, i.e., before or during SLD. The NMDA/4-AP model can, therefore, represent a valuable tool to gain insights into the role of specific cell populations in seizure generation, propagation and cessation.

## Highlights

• We propose a model of seizure-like events in temporal cortex slices from young mice.
• In the model local NMDA stimulations induce multiple seizure-like events.
• Electrophysiological, Ca^2+^ imaging and optogenetics are combined in the model.
• Seizures generation and propagation are separately studied.
• The role of specific cell population on these events can be accurately analyzed.

## 1 Introduction

Epilepsy is a severe neurological disorder characterized by recurrent convulsive or non-convulsive episodes reflecting massive neuronal discharges. Medial temporal lobe epilepsy (MTLE) is one of the most frequent and severe form of epilepsy in adulthood with seizures originating in hippocampal and para-hippocampal regions. As nearly one third of TLE patients are unresponsive to currently available antiepileptic drugs, the need for novel therapeutic tools, possibly with lower side effects, is impelling. The development of new therapeutic strategies relies on our knowledge of the cellular, molecular and network events that lead to seizure maturation, for which experimental animal models are of crucial importance. The most common animal models of epilepsy make use of chemoconvulsants such as pilocarpine and kainic acid (for review see Curia et al., 2008, Levesque and Avoli, 2013). Both toxins are used in rodents to induce status epilepticus which is followed by spontaneous recurring seizures after a latent period recapitulating in many aspects human TLE. As the epileptic phenotype develops in vivo, these models are considered valuable to study epileptogenesis (for a recent review on animal models see Kandratavicius et al., 2014).

A different approach uses acute slice preparations from non epileptic brain tissue that can, under certain conditions, develop seizures-like events. For example, slice perfusion with reduced extracellular Ca^2+^, Mg^2+^-free solutions (Jefferys and Haas, 1982, Jefferys, 1995, Trevelyan et al., 2006), increased extracellular K^+^ (Bear and Lothman, 1993) or the addition of compounds like pentylenetetrazol (PTZ), *N*-methyl-d,l-aspartate (NMDA), tetanus toxin or GABA receptors antagonists, including penicillin, picrotoxin or bicuculline, all cause a dramatic increase in neural network excitability that leads to the generation of epileptiform activity (Pinto et al., 2005, Pitkänen et al., 2006). It is noteworthy that these experimental conditions represent models of acute seizures generation (i.e., ictogenesis) rather than models of epileptogenesis. An additional diffused model of ictogenesis makes use of the K^+^ channel blocker 4-aminopyridine (4-AP) that induces epileptiform activity in brain slices in vitro (Voskuyl and Albus, 1985, Perreault and Avoli, 1991, Perreault and Avoli, 1992, Avoli et al., 1996, Ziburkus et al., 2006, Uva et al., 2009) and in vivo (Mihaly et al., 1990, Levesque and Avoli, 2013). In this model, after prolonged perfusion of the drug, seizure-like ictal discharges arise spontaneously at unpredictable time and locations (Avoli et al., 1996), but mainly in deep cortical layers (Avoli et al., 2002, Uva et al., 2009).

To study the abnormal activity that at the level of local circuits precedes the generation of focal seizures, as well as the dynamics of the cellular and network events that govern seizure propagation, it is necessary to identify the site of seizure initiation. However, the unpredictable nature of seizures renders the identification of this focal site a daunting challenge, especially in the intact brain. We describe here an experimental model in slice preparations from the entorhinal or temporal cortex of young rats and mice in which seizure-like discharges (SLDs) can be focally induced by challenging small groups of neurons with local NMDA applications. We will provide the experimental procedures in detail and discuss the applicability of the model in specific brain areas. As we show here, NMDA-evoked focal SLDs are indistinguishable from spontaneous events and their site of origin and propagation can be analyzed with unprecedented spatial and temporal accuracy. In the model, single and dual cell patch-clamp recordings, local field potential recordings, Ca^2+^ imaging and optogenetic techniques can be combined to provide insights into the cellular events that govern initiation and spread of SLDs. Explicative experiments are reported.

## 2 Methods

### 2.1 Animals

All experiments are carried out in strict accordance with the guidelines established by the European Communities Council Directive and approved by the National Council on Animal Care of the Italian Ministry of Health. All efforts are done to reduce the number of animals used. Brain slices (see Section 2.2) were obtained from Wistar rats, C57BL6J mice, G42 mice (Chattopadhyaya et al., 2004) (kindly donated by Alberto Bacci), and from GCaMP3::Pv-Cre mice obtained by crossing GCaMP3 mice (B6;129S-Gt(ROSA)26Sortm38(CAG-GCaMP3)Hze/J) with B6.Cg-Pvalbtm1.1(cre)Aibs/J mice (id #012358; Jackson Laboratory).

### 2.2 Brain slice preparations and Ca^2+^ dye loading

Coronal slices are prepared from postnatal day 14–20 animals as previously described (Cammarota et al., 2013). Briefly, rats and mice are deeply anaesthetized with intraperitoneally injected Zoletil (40 mg/kg) and decapitated; the brain is quickly removed and transferred to ice-cold standard ACSF, sACSF (in mM 125 NaCl, 2.5 KCl, 2 CaCl_2_, 1 MgCl_2_, 25 glucose, pH 7.4 with 5% CO_2_/95% O_2_). After brain dissection on the coronal plane, 350 μm-thick slices are cut with a vibratome (VT1000S, Leica Microsystems, GmbH, Wetzlar, Germany). Cutting solution for rat and mice brain are described in Cammarota et al. (2013) and in Dugue et al. (2005), respectively. Slices from mice are transferred for 1 min in a 95% O_2_ and 5% CO_2_ saturated solution containing (in mM) 225 d-mannitol, 2.5 KCl, 1.25 NaH_2_PO_4_, 26 NaHCO_3_, 25 glucose, 0.8 CaCl_2_, 8 MgCl_2_, 2 kynurenic acid with 5% CO_2_/95% O_2_. Slices from both rats and mice are finally transferred in sACSF at 30 °C for 20 min and then maintained at room temperature. Slices are kept in ACSF with Sulforhodamine 101 (SR-101) (0.2 μM, Sigma Aldrich, Milano) at 30 °C for 15 min to selectively stain astrocytes. Loading with the fluorescence Ca^2+^ indicator Oregon Green BAPTA1-AM (10 μM; Life Technologies, Monza) is performed at 30 °C for 50–60 min in the sACSF solution containing pluronic (0.12%, Sigma Aldrich, Milano, Italy) and kynurenic acid (1 mM, Sigma Aldrich, Milano, Italy). After loading, slices are recovered and kept at room temperature.

### 2.3 Ca^2+^ imaging

Images are acquired with a single-photon upright laser-scanning microscope (TCS-SP5-RS, Leica Microsystems, GmbH, Wetzlar, Germany) with an acquisition time frame of 491 ms (seven line averaging). Oregon Green and GCaMP3 are excited at 488 nm, SR-101 at 543 nm. The microscope is also equipped with a CCD camera for differential interference contrast (DIC) image acquisition.

### 2.4 Electrophysiology and induction of focal SLDs

Brain slices are continuously perfused in a submerged chamber (Warner Instruments, Hamden, USA) at a rate of 3–4 ml min^−1^ with (in mM): NaCl, 120; KCl, 3.2; KH_2_PO4, 1; NaHCO_3_, 26; MgCl_2_, 0.5; CaCl_2_, 2; glucose, 10; at pH 7.4 (with 5% CO_2_/95% O_2_). Single and dual cell recordings are performed in current-clamp and voltage-clamp configuration using a multiclamp-700B amplifier (Molecular Devices, Foster City, CA, USA). Signals are filtered at 1 kHz and sampled at 10 kHz with a Digidata 1440s interface and pClamp10 software (Molecular Devices, Foster City, CA, USA). Typical pipette resistance was 3–4 MΩ. Access resistance is monitored throughout the recordings and was typically <25 MΩ. Whole-cell intracellular pipette solution is (in mM): K-gluconate, 145; MgCl_2_, 5; EGTA, 0.5; Na_2_ATP, 2; Na_2_GTP, 0.2; HEPES, 10; to pH 7.2 with KOH, osmolarity, 280–300 mOsm (calculated liquid junction potential: −14 mV). All patched neurons are from cortical layer V–VI; pyramidal neurons are voltage-clamped at −50 mV (Vh) or current clamped at resting potential as with Pv-FS interneurons. Induction of focal SLDs is performed in the presence of 4-AP (100 μM) and bath temperature maintained at 30–32 °C by an inline solution heater and temperature controller (TC-324B, Warner Instruments, Hamden, USA). A pressure ejection unit (PDES, NPI Electronics, Tamm, Germany) is used to apply a single or double pulse to NMDA (1 mM, Sigma Aldrich, Milano, Italy)-containing pipettes with a 3 s interval, a pressure of 4–10 psi, and a duration of 300–600 ms. In some experiments, the NMDA glass pipette includes an AgCl_2_ electrode for extracellular local field potential recordings. Field potential signals are filtered at 1 kHz, amplified by an AM-amplifier (AM-systems, Carlsborg, WA, USA) and sampled at 10 kHz.

### 2.5 Data analysis

In voltage-clamp recordings, the recruitment of principal neurons into propagating ictal events is marked by the transition from the predominant inhibitory to the predominant excitatory phase (*t*_IE_), which is calculated as described in Cammarota et al. (2013). Ictal event duration in voltage-clamp recordings is calculated from the *t*_IE_ to the end of the last afterdischarge recorded with an instant frequency higher than 0.1 Hz. Spectrogram of local field potential recording are performed with an algorithm written in Matlab using logarithmic wavelet windows. The Ca^2+^ signal is reported as Δ*F*/*F*_0_, where *F*_0_ is the baseline fluorescence. Significant Ca^2+^ events are considered when exceeding three folds SD of baseline. Data analysis was performed with Clampfit 10 (Molecular Devices, Foster City, CA, USA), Origin 8.0 (Microcal Software), Leica Application Suite (Leica Microsystems, GmbH, Wetzlar, Germany), Microsoft Office and MATLAB 7.6.0 R2008A (Mathworks, Natick, MA, USA).

## 3 Results

### 3.1 Focal ictal discharges in rat temporal and entorhinal cortex

**Fig. 1:**
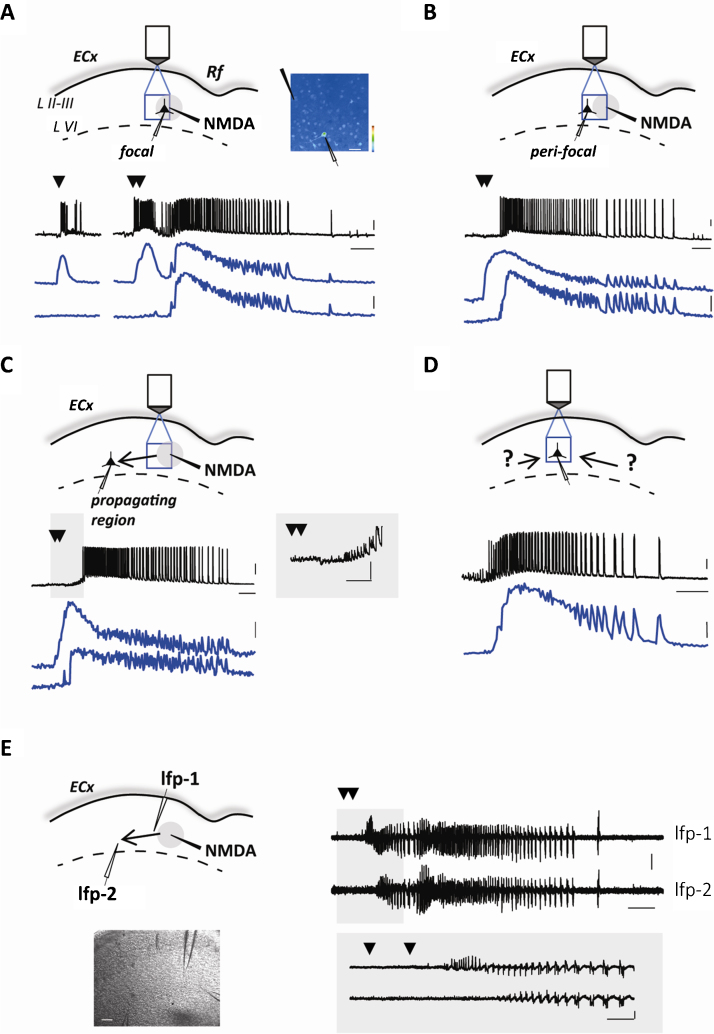
Focally induced SLDs in entorhinal cortex slice preparations from young rats. (A–C). Upper panels, schematic of the experiments with the NMDA pipette (black), the focal area (gray circle), the patched pyramidal neuron and the area of Ca^2+^ imaging (blue square) in rat entorhinal cortex. Lower traces, membrane potential of the pyramidal neuron (black traces) and simultaneous average Ca^2+^ signal from 4 putative pyramidal neurons located in the focal (upper blue traces) and peri-focal (lower blue traces) area. In A, right panel reports the Oregon-Green BAPTA-1 fluorescence with indication of the NMDA and the patch pipette (black and white, respectively). (D) Schematic of the experiment with patch recording from a pyramidal neuron and simultaneous average Ca^2+^ signal from 3 putative pyramidal neurons located in the same region during a spontaneous SLD. Scale bars: 20 mV; 20% Δ*F*/*F*_0_; 10 s (5 s for inset in (C)). Black arrowheads in this and other figures mark the NMDA pulses. E. Left panel, schematic of the experiment with two electrodes for local field potential (lfp) recording, one in the peri-focal region (250 μm) and the other distant 550 μm form the NMDA-pipette tip, and DIC image (scale bar 100 μm) of the rat brain slice during the experiment. Right traces, LFP recordings during the evoked SLD. Scale bars, 0.1 mV; 10 s. (For interpretation of the references to color in this figure legend, the reader is referred to the web version of this article.)

Whole-cell patch-clamp recordings from single or dual neurons and simultaneous Ca^2+^ imaging from different neuronal cells and glia in layer V of entorhinal or temporal cortex slices from young rats (PN 15-P19) can provide useful indications on the generation and spread of epileptic seizure-like discharge (SLD) at the level of both individual neurons and network provided that the site and the time of SLD initiation can be predicted. This is achievable in cortical slice preparations perfused with 4-AP (50–100 μM) and reduced Mg^2+^ (0.5 mM). After 10 min of slice perfusion, largely before spontaneous epileptiform activity could emerge, brief pressure pulses (4–10 psi; 300–600 ms) are applied to a NMDA containing glass pipette (NMDA: 1 mM; typical tip resistance, 2 MΩ), located in layer V in proximity to the rhinal fissure, 20–30 μm below the slice surface (Fig. 1A), every 4–5 min. Single NMDA pulses evoke only a transient (10–20 s) membrane depolarization and action potential firing in a current-clamped pyramidal neuron located at ∼150 μm from the NMDA-pipette tip and simultaneous Ca^2+^ elevations in other neighboring neurons. With respect to single NMDA pulses, double NMDA pulses cause a larger membrane depolarization and a more intense firing in the patched neuron as well as Ca^2+^ elevations in a larger number of neurons. The direct neuronal response to double NMDA pulse defines a region of about 200 μm in radius, that we named focal area (gray circles in this and the other figures). Most importantly, the response to double NMDA pulses is followed by a SLD typically characterized by a tonic phase of sustained depolarization followed by a clonic phase of rhythmic bursting events, i.e., the afterdischarges, that according to the accompanied Ca^2+^ signal occur in the neuronal network with a high degree of synchrony (Fig. 1A). Notably, neurons located immediately outside the focal region (a corona between 200 and 300 μm from the NMDA-pipette tip that we termed peri-focal region), exhibit Ca^2+^ elevations (lower blue traces) that match those of neurons in the focal region (upper blue traces), with the only exception of the initial Ca^2+^ increase induced directly by NMDA (Fig. 1A–C). The Ca^2+^ elevation directly activated by NMDA in neurons located at the focus often hampers the possibility to identify from these neurons the timing for SLD onset (see upper Ca^2+^ traces in Fig. 1B and C) which can be more clearly and precisely evaluated from the response of neurons in the peri-focal region (Fig. 1A–C). After double NMDA pulses, SLDs are generated with a delay that marks the transition from a focal hyperexcitable network to a fully propagating epileptiform event. Depending on the strength of local circuit inhibition (see below), this delay can be as brief as a few seconds. In general, it corresponds to about 10 s (mean, 10 ± 1 s with respect to the first of the two NMDA pulses, range 5–18 s; *n* = 127 SLDs, 18 rats). A double NMDA pulse (3 s apart) successfully evokes an ictal discharge in 82% of the trials (*n* = 162 SLDs out of 198 trials, 18 rats). In contrast, a single NMDA pulse (Fig. 1A) is successful in only 15% of the trials (*n* = 5 SLDs out of 32 trials, 18 rats).

The SLD initiation can be studied by Ca^2+^ imaging from the focal and peri-focal regions while the lateral propagation of SLDs across layer V temporal cortex can be monitored by recording from a pyramidal neuron located in the propagating region, i.e., 800–1000 μm from the NMDA-pipette tip. As shown in Fig. 1C, NMDA-evoked SDLs propagated to the distant neuron with a mean delay of 21 ± 1 s (*n* = 28 SLDs from 11 experiments, 8 rats). In particular, SLD propagation was characterized by a series of hyperpolarizations that preceded the full recruitment of the distant pyramidal neuron (Trevelyan et al., 2006, Trevelyan et al., 2007, Cammarota et al., 2013). Notably, the SLDs recorded in pyramidal neurons from the peri-focal or from the propagating region are undistinguishable from spontaneous events. These events are only occasionally observed in these experiments probably because eliciting multiple SLDs by repetitive NMDA applications prevents the occurrence of further spontaneous SLDs. Spontaneous events can reliably be observed to arise from unknown foci in specifically designed experiments in which NMDA stimuli were not applied.

The dynamics of NMDA-evoked SLDs can be also monitored by extracellular local field potentials (LFP) recorded through glass pipettes that can be differently positioned with respect to the focus. In LFP recordings, SLDs are characterized by initial high frequency, low voltage signals followed by large amplitude low frequency events that parallel the clonic phase of the afterdischarges. In Fig. 1E we report an example of two LFPs from peri-focal (LFP-1) and distant region (LFP-2) which illustrates the generation and the delayed propagation of SLDs. By outlining the typical pattern of epileptiform events commonly observed in different in vitro and in vivo models of epilepsy, LFP recordings further validate our experimental model of focal SLDs.

### 3.2 Focal seizure-like discharges in the mouse entorhinal and temporal cortex

**Fig. 2:**
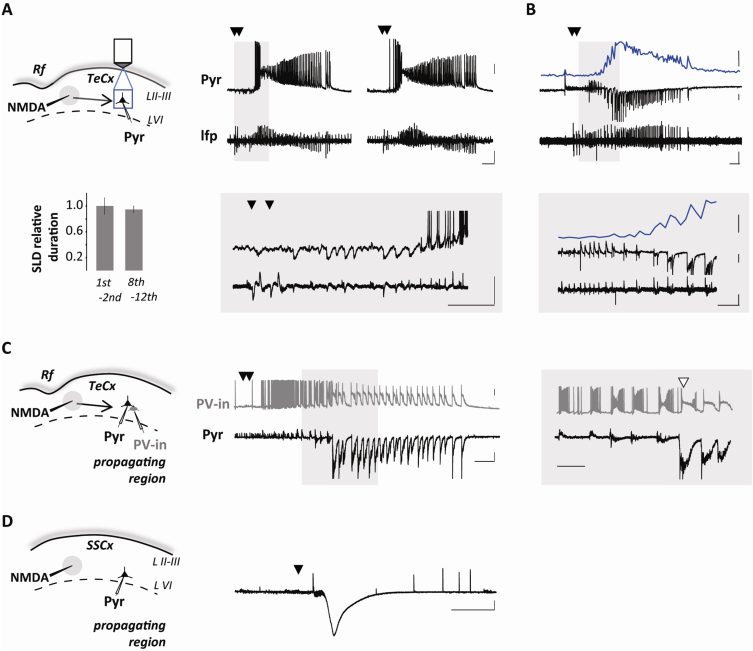
Focally induced SLDs in temporal cortex slice preparations from young mice. (A, C and D) Left panels, schematic of the experiments in mouse temporal cortex (in somatosensory cortex for (D)) with the NMDA pipette (black), also used for lfp recording in A, the focal area (gray circle), the patched pyramidal neuron (and a Pv-interneuron, (C)) in the propagating region located 1000 μm from the focal area and the region of Ca^2+^ imaging (blue square). (A) Right traces, membrane potential of the pyramidal neuron and lfp recording during 2° and 8° evoked SLDs and histogram showing the relative duration of successive NMDA evoked SLDs (average of the 1st and 2nd event vs average of 8th to 12th events). (B) Whole-cell current of the pyramidal neuron (middle black trace, Vh = −50 mV), lfp recording (lower black trace) and average Ca^2+^ signal from 3 putative pyramidal neurons (blue traces) located in the same propagating region as in (A) Scale bars, 20 mV; 0.1 mV; 200 pA; 20% Δ*F*/*F*_0_; 10 s; for lower insets, 5 s. (C) Right traces, membrane potential of the PV-interneuron (PV-in; gray trace) and simultaneous whole-cell current of the pyramidal neuron (Vh −50 mV, black trace) in slice from a G42 mouse. White arrowhead in inset marks depolarization block in the PV-interneuron and full excitatory input in the pyramidal neuron. Scale bars, 20 mV, 200 pA, 5 s; for right inset 2 s. (D) Right trace, whole-cell current of the pyramidal neuron (Vh −50 mV) showing a cortical spreading depression like event. Scale bars, 500 pA, 2 min. (For interpretation of the references to color in this figure legend, the reader is referred to the web version of this article.)

Over the last two decades transgenic and gene targeting techniques combined with optogenetics resulted in a large number of genetically modified mouse strains that have been used to define the role of distinct neuronal populations in brain functions. Furthermore, these techniques have been proved to be extremely useful for improving our understanding of the pathological mechanisms at the basis of brain disorders including epilepsy. It is, therefore, important to further characterize our model of focal epilepsy in the mouse brain and extend it to other brain regions, such as the temporal cortex. We here reveal that in slices from the temporal cortex of young C57BL6J mice (PN 16-22), a single NMDA pulse applied in the presence of 4-AP and low extracellular Mg^2+^ (0.5 mM) is poorly successful in evoking a SLD (only 15% successes; 9 of 61 trials; 24 mice) with respect to double NMDA pulses (92% successes, 117 of 127 trials; 24 mice). Successive double NMDA pulses evoke SLDs with reproducible pattern and duration (Fig. 2A; mean duration in the entorhinal cortex, 77 ± 7 s, *n* = 24 SLDs from 10 experiments, 4 mice; mean duration in the temporal cortex, 69 ± 4 s, *n* = 43 SLDs from 6 experiments, 5 mice), provided that an interval of at least 4–5 min is used to allow the recovery from post-seizure refractory periods. Notably, the same NMDA-containing pipette which delivers NMDA can be used as an extracellular electrode to monitor at the focus the generation of evoked SLDs (see Section 2; Fig. 2A). NMDA-evoked SLDs propagate to distant pyramidal neurons after a series of hyperpolarizing events (Fig. 2A, insets). In voltage-clamp recordings at −50 mV, these events correspond to large outward inhibitory currents generated by GABAergic interneurons that delay the progression of SLDs (Trevelyan et al., 2006, Trevelyan et al., 2007) (Fig. 2B). As we reported (Cammarota et al., 2013), the recruitment process occurs subsequently to the collapse of the feedforward inhibition and it is marked by both the transition from inhibition to excitation in the voltage-clamp recordings from pyramidal neurons and the intracellular Ca^2+^ elevations in these cells. Pyramidal neurons in the peri-focal region are recruited into the propagating SLD with a mean delay of 11 ± 1 s (*n* = 33 SLDs,) after the double NMDA pulse. At 1 mm from the NMDA-pipette this delay increases to 21 ± 1 s in the entorhinal cortex (*n* = 24 SLDs, 10 experiments, 4 mice) and 21 ± 2 s in the temporal cortex (*n* = 12 SLDs, 6 experiments, 4 mice). The use of dual patch-clamp recordings in the NMDA/4-AP model allows to study how the activity of a specific interneuron class can affect SLD propagation. As an example, dual recordings from a parvalbumin-expressing GABAergic interneuron and a neighboring pyramidal neuron in slices from G42 mice show that inhibitory barrages preceding SLD in the pyramidal neuron are temporally correlated with the firing activity in the parvalbumin interneuron. These interneurons have been shown to represent a main source of feedforward inhibition and only after their firing discharge suddenly enter into a depolarization block are the neighboring pyramidal neurons fully recruited into the propagating SLD (Cammarota et al., 2013) (Fig. 2C).

In contrast to entorhinal and temporal cortex from rats, both entorhinal and temporal cortex from C57BL6J mice showed to be less prone to seizure generation. In particular, we found that 20% (*n* = 3 out of 15 slices; 10 animals) and 27% of mouse brain slices (15 out of 55 slices; 28 animals) were completely refractory to seizure generation in entorhinal and temporal cortex, respectively, with respect to only 8% of rat (*n* = 15 out of 199 slices; entorhinal and temporal cortices pooled data; 110 animals).

The somatosensory cortex (SCx) from C57BL6J mouse appears fully refractory to NMDA-induced SDLs. In SCx slices obtained from PN16-18 mice, upon recordings from layer V pyramidal neurons (5 experiments, 3 animals) we failed to observe focal SLDs after performing 30 double NMDA pulse stimulations. Following 4 of these trials we observed instead a prolonged and sustained depolarization in the pyramidal neurons that closely resembles a typical cortical spreading depression (CSD) (Fig. 2D).

### 3.3 Cellular players during focal ictal discharge generation: Neurons and astrocytes

**Fig. 3:**
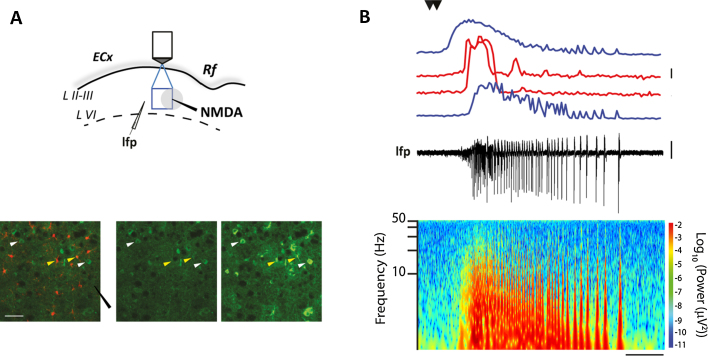
Astrocyte and neuron activity during SLD generation. Left panel, schematic of the experiment in a mouse entorhinal cortex slice with the NMDA pipette (black), the focal area (gray circle), the LFP electrode positioned 300 μm from the NMDA pipette and the area of Ca^2+^ imaging (focal and peri-focal region, blue square). Left lower panels, images of the GCaMP3 and SR-101 fluorescence (green and red, respectively) at basal (left and middle panels) and during the NMDA-evoked SLD (right panel). Scale bar, 50 μm. Right, simultaneous lfp recording (black trace) and Ca^2+^ signal from neurons in the focal (upper blue trace) and perifocal area (lower blue trace) and from astrocytes from the focal region (red traces). Lower panel, spectrogram of the recorded lfp. Scale bars, 0.01 mV, 50% Δ*F*/*F*_0_; 10 s. (For interpretation of the references to color in this figure legend, the reader is referred to the web version of this article.)

Knowing where a SLD will be generated provides the unique opportunity to study the abnormal activity that at the level of local circuits precedes SLD generation. To this aim, Ca^2+^ imaging from different neurons and glia can provide important information on network activities at the focus. As an explicative example, we illustrate in Fig. 3 the use of GCaMP3, a genetically encoded calcium indicator (GECI) (Tian et al., 2009). Although GECI can be selectively expressed in specific cell populations with the Cre recombinase system, due to an unspecific Cre recombinase expression in this mouse line, GCaMP3 results to be present in a number of interneurons, pyramidal neurons and astrocytes (see Section 2, Fig. 3A). In Fig. 3B, a typical NMDA-evoked SLD from the entorhinal cortex of these mice is reported by simultaneous LFP and Ca^2+^ signal recordings from different cell types. The spectrogram shows the typical pattern of an epileptiform event with rhythmic activity in the beta-gamma frequencies. Astrocytes, that are selectively marked with sulforhodamine 101, show large Ca^2+^ transients that differ substantially from neuronal ones. In the focal region, a group of astrocytes exhibit large amplitude Ca^2+^ elevations (red traces) immediately after the direct activation of neurons by NMDA (upper blue trace) and before the SLD initiation that is revealed from the Ca^2+^ signal changes in the peri-focal neurons (lower blue trace). As we previously reported (Gomez-Gonzalo et al., 2010), these early activated astrocytes contribute to lower the threshold of NMDA-evoked SLDs probably through the release of gliotransmitters promoting neuronal synchrony.

## 4 Discussion

We here describe an experimental model of focal epileptiform activity in cortical slices from young rats and mice. In this model, a focal SLD is generated after a transition phase that follows an episode of intense activity induced in a group of layer V neurons by a local NMDA application in the presence of 4-AP and low Mg^2+^. With the use of different combinations of single-dual cell patch-clamp recordings with local field potential and Ca^2+^ imaging techniques, we here extend to the temporal cortex of rats and mice the model that was originally developed in the rat entorhinal cortex.

Although a different susceptibility to epileptiform activity is observed in rats and mice, with slices obtained from mice being less prone to SLD generation with respect to slices from rats, both species can be used with this model that represents a powerful tool to study with high accuracy the spatial and temporal features of seizure generation and propagation. Indeed, as we already described in the rat entorhinal cortex (Gomez-Gonzalo et al., 2010, Losi et al., 2010), also in mouse entorhinal and temporal cortex multiple SLDs can be evoked by repetitive NMDA challenges and the evoked SLDs exhibit similar pattern and duration. As discussed below, the use of mice has the great advantage, with respect to rats, to exploit different transgenic lines for identification and selective stimulation of specific cell populations.

We also show that as opposed to entorhinal and temporal cortex, NMDA local applications in a different cortical area, such as the somatosensory cortex, fail to induce SLDs, and occasionally evoke cortical spreading depression-like events.

The NMDA/4-AP model can be used to gain insights into the cellular events at the basis of SLD generation and propagation. We can study how the activity of specific cell populations, from either the focus or the propagating region, evolves during the different phases of a SLD, including the transition phase, the development of tonic/clonic discharges and the cessation. Because multiple SLDs initiating from the same focal site can be induced, we can also analyze whether and how these distinct activities can eventually change upon the recurrent impact of the successive SLDs. Furthermore, using optogenetic tools, a specific class of neurons or interneurons can be selectively activated or inhibited by non-invasive light pulses delivered to a spatially defined location, i.e., the focus or the propagating region, and at a precise time, i.e., before or during the different SLD phases. For example, we can modify the response of the neural network to NMDA, possibly lowering or increasing the threshold of SLD generation. Changes in SLD threshold can be, indeed, evaluated in the model given that two successive NMDA pulses, as opposed to a single NMDA pulse at the same intensity and duration, are needed to successfully evoke a SLD. In the model, single and double NMDA pulses can represent, therefore, sub- and supra-threshold stimuli, respectively, which can be used to study different conditions or drug treatments that favor or oppose SLD generation. It is note worth that the threshold of SLD generation appears to slightly decrease along with prolonged 4-AP perfusion. This should be taken into account in studies aimed to evaluate the conditions that eventually favor SLD generation. One obvious way to overcome this caveat is to perform as control two sub-threshold NMDA stimulations, one before and the other *after* the experimental conditions that lower SLD threshold. Using this model, we could reveal that activation of astrocytes at the focus favors SLD generation by lowering SLD threshold, while the inhibition of Ca^2+^ elevations in these cells increases SLD threshold (Gomez-Gonzalo et al., 2010). Recently, using the NMDA/4-AP model we could reveal a functional dichotomy of Parvalbumin-positive (Pv) inhibitory interneurons in epileptiform activity. We found that an optogenetic activation at the focus of Pv interneuron expressing the light-gated cation channel channelrhodopsin-2 (Zhang et al., 2010) enhances the generation of SLDs, while a similar activation restricted to Pv interneurons distant from the focus blocked SLD propagation (Sessolo, Marcon et al., J Neurosci, in press).

An important advance in optogenetic techniques is the use of patterned illumination through light phase modulation that allows the selective activation/inhibition of a few cells as opposed to classical illumination methods (Dal Maschio et al., 2010, Bovetti and Fellin, 2015). In the NMDA/4-AP model this approach would allow to functionally test, for instance, whether activation or inhibition of a defined number of cells in the focal region is sufficient to favor or oppose SLD generation.

In conclusion, as we move forward in our attempts to understand the mechanism(s) of epilepsy, it is important to identify models of focal epileptiform activity in vitro and in vivo that combined with modern optogenetics (Zhang et al., 2010, Tye and Deisseroth, 2012, Kokaia et al., 2013, Packer et al., 2013) and GECI have the potential to provide fundamental insights into the different role played by specific cell populations in seizure generation, propagation and cessation.
